# Hashimoto’s Thyroiditis in Noonan Syndrome: A Case Report

**DOI:** 10.7759/cureus.51592

**Published:** 2024-01-03

**Authors:** Qaisar Ali Khan, Yaxel Levin-Carrion, Rohail Khan, Aleena Z Khan, Sumaira Saddiq, Vaishnavi Guddeti, Adithya Nadella, Amritpal Kooner, Ayiz Jan, Ameer M Farrukh

**Affiliations:** 1 Internal Medicine, Khyber Teaching Hospital, Peshawar, PAK; 2 Internal Medicine, District Headquarter Hospital KDA Kohat, Kohat, PAK; 3 Medicine, Rutgers University New Jersey Medical School, Newark, USA; 4 Emergency Medicine, Shifa College of Medicine, Islamabad, PAK; 5 Medicine, Islamabad Medical & Dental College, Islamabad, PAK; 6 Medicine, Khyber Teaching Hospital, Peshawar, PAK; 7 Internal Medicine, Nanjing Medical University, Nanjing, CHN; 8 Medicine, Chicago College of Osteopathic Medicine, Chicago, USA; 9 Medicine, Saidu Medical College, Saidu Sharif, PAK; 10 Medicine, University of Galway, Galway, IRL

**Keywords:** hashimoto’s thyroiditis, karyotyping, facial dysmorphism, hypothyroidism, noonan syndrome

## Abstract

Noonan syndrome is a genetic, developmental disorder characterized by facial deformities, congenital heart defects, webbed neck, wide space nipples, and growth hormone deficiencies. We report a case of a 15-year-old female patient who presented to the outpatient department with recurrent puffiness of both eyes, easy fatiguability, and dyspnea on exertion. The condition was associated with bilateral proximal muscular weakness of lower limbs with positive Gower’s sign. On examination, the patient had a webbed neck, hypertelorism, a shielded chest, short stature, and a high-arched palate. Thyroid function tests revealed hypothyroidism. Chromosomal analysis revealed 46 XX. After excluding Turner syndrome on karyotyping, Noonan syndrome with hypothyroidism was diagnosed. The patient was started on levothyroxine and referred to a pediatric endocrinologist for further growth and development assessment.

Autoimmune hypothyroidism in a patient with Noonan Syndrome is rare; it may occur as a separate entity or have some genetic susceptibility. Further research is needed to determine the association of autoimmune hypothyroidism with Noonan syndrome.

## Introduction

Noonan syndrome, a multisystemic, genetic, developmental disorder, has an estimated incidence of one per 1000 to one per 2500 live births in the United States [1]. Pediatrician and heart specialist Jacqueline Noonan and Ehmke described a series of patients with unique facial features and multiple malformations, including congenital heart defects, and they subsequently coined the term "Noonan syndrome." to refer to the condition [2]. The condition shares some characteristic abnormalities with Turner syndrome, which only affects females, leading to its former label as "male Turner syndrome." Nevertheless, the term is now outdated as Noonan syndrome is known to affect both genders. The disorder is also called webbed neck syndrome, pseudo-Ullrich Turner syndrome, female pseudo-Turner syndrome, or Turner-like syndrome [3]. Clinically, Noonan syndrome is mainly characterized by a range of features, including facial dysmorphism, congenital heart defects, growth hormone deficiencies, webbed neck, wide-spaced nipples, and musculoskeletal, renal, genital, and bleeding abnormalities. Additionally, about 25% of patients with Noonan syndrome may experience intellectual developmental disorders. Facial abnormalities, such as hypertelorism, down-slanting eyes, webbed neck, eyelid abnormalities, and skin manifestations, further contribute to these individuals' characteristic appearance. Prenatal presentation of Noonan syndrome may involve complications like polyhydramnios, fetal edema, increased nuchal translucency, and cystic hygroma [4].

The autoimmune nature of Hashimoto's thyroiditis suggests complex interactions between genetic predisposition, environmental triggers, and immune dysregulation. In the context of Noonan syndrome, which involves mutations in genes affecting RAS-MAPK signaling, there may be a potential association between the dysregulated pathway and the development of autoimmune thyroiditis. This intriguing link indicates the possibility of shared genetic susceptibility factors or disrupted immune tolerance mechanisms, warranting in-depth evaluation of autoimmune thyroid function in patients with Noonan syndrome [5]. Despite the extensive research on Noonan syndrome, information regarding its association with hypothyroidism remains scarce, underscoring the need to further investigate the potential link between these two conditions. This case report highlights the concomitant occurrence of hypothyroidism in a patient with Noonan syndrome, calling for greater attention to be paid to this aspect of the syndrome.

This article was previously posted to Authorea preprint on June 20, 2023. This case was reported based on the SCARE 2020 guidelines [6].

## Case presentation

**Figure 1:**
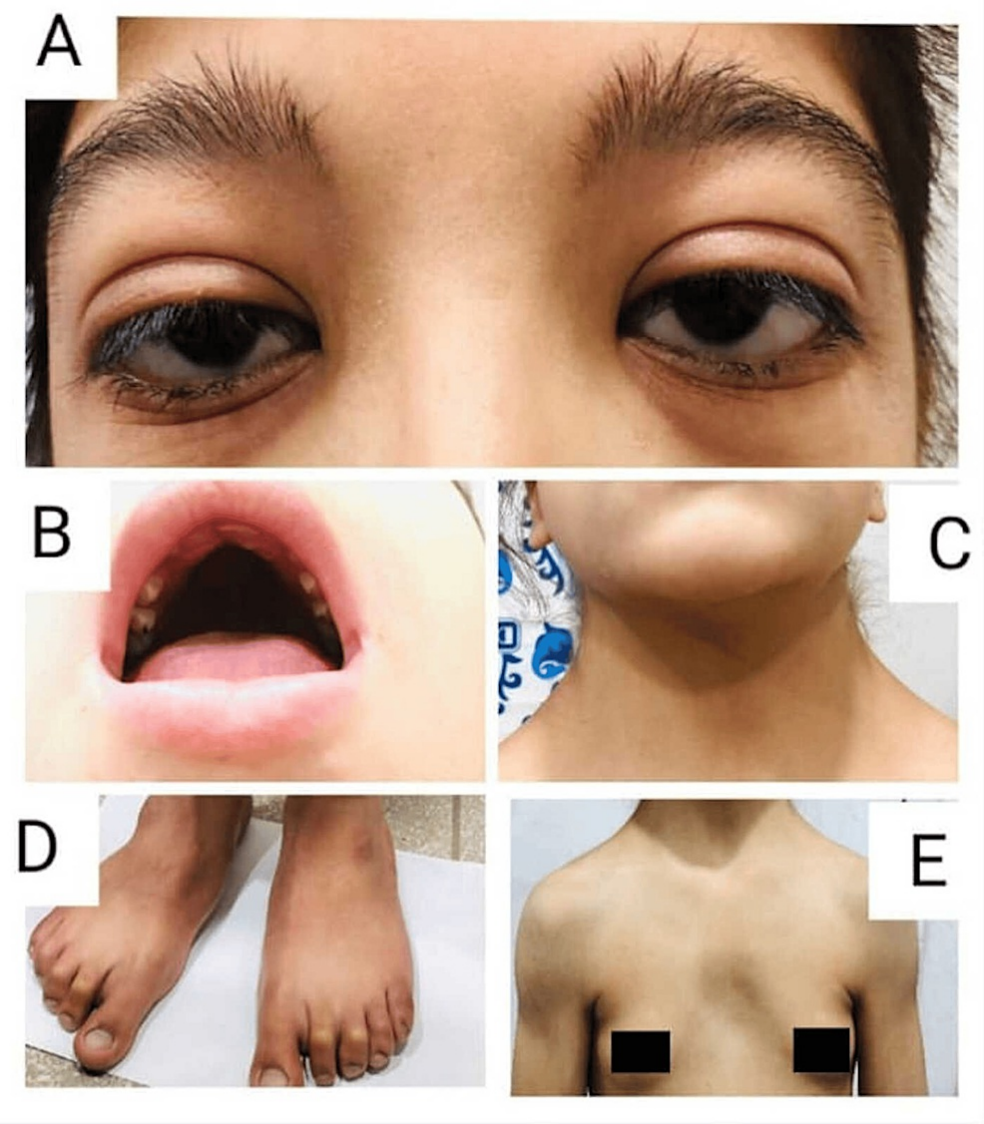
The images show down-slanting eyes, hypertelorism (A), high-arched palate (B), webbed neck (C), flat feet (D), widely spaced nipples, and shield chest (E)

A 15-year-old female patient presented to the outpatient department of a tertiary care hospital due to bilateral periorbital puffiness, easy fatiguability, and generalized body weakness. The patient's condition had started insidiously six months prior, and it had gradually worsened. Further inquiry revealed a history of constipation on and off that was relieved with laxatives. The patient also had facial deformities, short stature, and a webbed neck since birth. The patient had been born at term through normal vaginal delivery at the hospital. The patient's mother had an uneventful antenatal history. The patient showed no evidence of significant organ defect or deformity at birth. She had received all the childhood vaccinations, and all developmental milestones had been achieved on time. Family history was nonsignificant for congenital heart defects, intellectual disability, short stature, or unusual facial features. She was 135 cm (below 5 percentile) tall, weighed 32 kg (below the fifth percentile), and had a blood pressure of 100/70 mmHg, pulse rate of 65 beats per minute, and respiratory rate of 15 breaths per minute. Examination revealed down-slanting eyes, hypertelorism, webbed neck, and shield chest with wide-spaced nipples, as shown in Figure 1.

Systemic examination was unremarkable. Laboratory tests were ordered, as shown in Table 1.

**Table 1:** Laboratory investigations MCV: mean corpuscular volume; TSH: thyroid-stimulating hormone

Laboratory test	Results	Normal range
Total leukocyte count	5.56 x 10^3^/uL	4-11
Hemoglobin	9.3 g/dL	11.5-17.5
MCV	76 fL	80-100
Platelets count	165 x 10^3^/uL	150-450
Serum ferritin	35 ng/mL	25-200
Alanine transaminase	23.8 mg/dl	10-50
Total bilirubin	0.35 mg/dl	0.1-1.0
Alkaline phosphatase	74 U/L	<187
Serum albumin	4.0 g/dl	3.4-5.2
Urea	22.5 mg/dl	10-50
Creatinine	0.85 mg/dl	0.3-0.8
Creatinine kinase	1417 U/L	26-140
Serum TSH	100 uIU/L	0.3-4.2
Free T4	1.48 pmol/L	10-28
T3 total	0.195 nmol/L	0.6-2.0
24-hour urinary protein	9.3 mg/dL	<10

**Figure 2:**
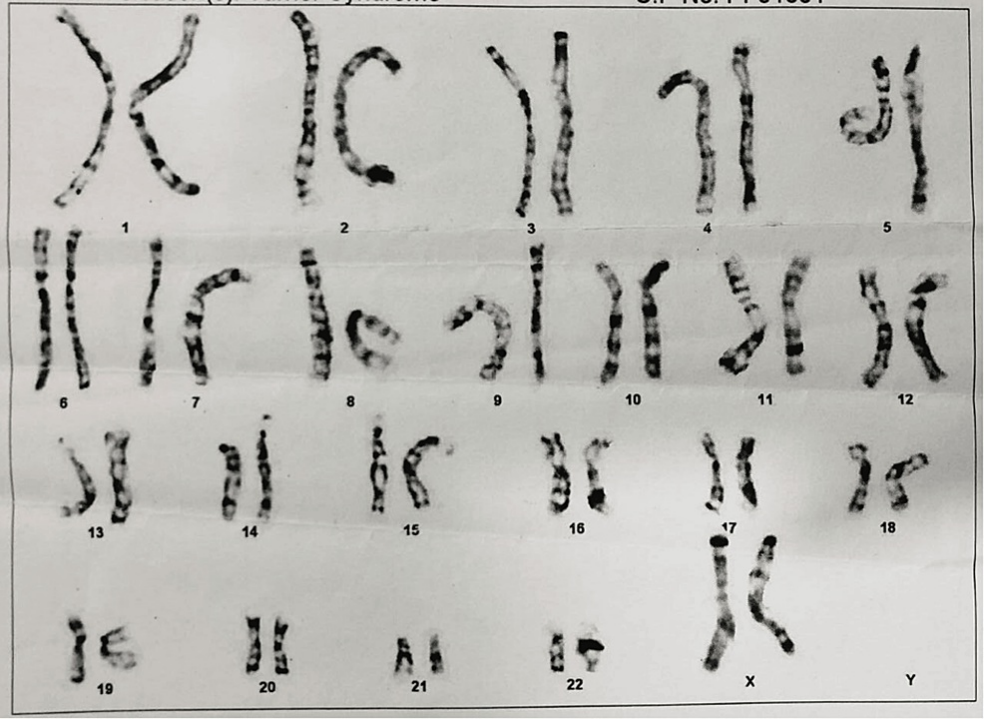
Karyotyping of the patient

Based on laboratory testing, autoimmune hypothyroidism was diagnosed as the cause of the patient's recurrent eye puffiness, easy fatiguability, and generalized body weakness. Her clinical features were suggestive of congenital syndrome, i.e., Turner syndrome or Noonan syndrome. Karyotyping of the patient showed 46 XX, as shown in Figure 2.

Genetic testing was not done for confirmation due to affordability issues; however, given the patient's clinical features and normal karyotyping, a presumptive diagnosis of Noonan syndrome was made. She was further screened for other conditions associated with Noonan syndrome; her echocardiography, coagulation profile, and abdominal ultrasonography were unremarkable. The patient and her parents were counseled about the condition; she was started on levothyroxine 50 mcg once daily, and an iron sulfate 200 mg capsule (one capsule daily for two months). She was referred to a pediatric endocrinologist for growth and development assessment. She was also instructed to follow up closely with repeat thyroid function tests after six weeks. At the follow-up visit, her symptoms had started to improve and her serum TSH came out 33 uIU/L. She was continued on levothyroxine.

## Discussion

Phenotypically, Noonan syndrome, especially in females, shares similar features with Turner syndrome, such as short stature, webbed neck, widely spaced nipples, intellectual disability, urogenital and cardiac abnormalities, and craniofacial dysmorphism. Karyotyping is the only method that enables us to differentiate between these two conditions [5]. Noonan syndrome is a genetic condition, and the common mutations involve PTPN11, SOS1, RAF1, KRAS, NRAS, and BRAF genes. Among these mutations, PTPN11 gene mutations account for about 50%, SOS1 gene mutation for 10-15%, and RAF1 gene mutation for 5-10% of Noonan syndrome cases. KRAS, NRAS, and BRAF genes account for a relatively small proportion of Noonan syndrome incidence [7,8]. Noonan syndrome can be diagnosed by genetic testing or clinical diagnostic criteria. The scoring system for the diagnosis of Noonan syndrome was developed by Van der Burgt et al. in 1997, and it comprises six major features and six minor features. According to these criteria, Noonan syndrome can be diagnosed based on typical facial features plus one major or two minor characteristics or suggestive facial features plus two major or three minor signs [5]. Our patient had typical facial features with one major criterion, i.e., short stature (below the third percentile). Table 2 shows the scoring system for Noonan syndrome.

**Table 2:** Noonan syndrome scoring system HOCM: hypertrophic obstructive cardiomyopathy; NS: Noonan syndrome

Features		
	A = major	B = minor
Facial	Typical face dysmorphology	Suggestive face dysmorphology
Cardiac	Pulmonary valve stenosis, HOCM, and/or ECG typical of NS	Other defects
Height	less than the third percentile according to age	less than the 10th percentile according to age
Chest wall	Pectus carinatum/excavatum	Broad thorax
Family history	First-degree relative with definitive NS	First-degree relative with suggestive NS
Other	Intellectual disability, cryptorchidism, and lymphatic dysplasia	One of the following: intellectual disability, cryptorchidism, lymphatic dysplasia

Differential diagnoses of Noonan syndrome include Turner syndrome (45, XO), cardiofaciocutaneous (CFC) syndrome, Costello syndrome, Neurofibromatosis type 1 (NF1), and LEOPARD syndrome. Syndromes characterized by facial dysmorphology, short stature, and cardiac defects may sometimes be difficult to differentiate from Noonan syndrome, notably Williams syndrome and Aarskog syndrome. As the syndrome is associated with a wide spectrum of disorders, patients with Noonan syndrome have to undergo hematological investigations, karyotyping and mutation analysis cardiac investigations, and assessment of development (IQ, identifying any delays, intellectual disability) [1,9].

A previous study has shown the association between hypothyroidism and Noonan syndrome although few studies report autoimmune thyroiditis; the cause of the condition is unknown, but factors that may predispose individuals to it include genetics, high iodine consumption, and age. A study by Vesterhus et al. reported that thyroid antibodies were found to be more common in Noonan syndrome, but hypothyroidism is not more common in Noonan syndrome compared to the general population [10,11]. There is no single, unique treatment available for Noonan syndrome, and hence a multidisciplinary approach is needed to address these patients' concerns and to treat the complications or associated conditions.

## Conclusions

Noonan syndrome is a genetic, developmental disorder that can affect any system of the body. Primary hypothyroidism in a patient with Noonan syndrome is a rare phenomenon, and it can occur either as a separate entity or might have an association with Noonan syndrome. A multidisciplinary approach focusing on patient concerns and treatment of associated conditions is required for favorable outcomes. Further research is needed to find out whether in vitro autoimmune Hashimoto's thyroiditis led to this congenital disorder or vice versa.
